# Solubility Constraints on Aquatic Ecotoxicity Testing of Anionic Surfactants

**DOI:** 10.1007/s00128-018-2361-1

**Published:** 2018-06-01

**Authors:** J. Hammer, A. M. Tukker, J. F. Postma, J. J. -H. Haftka, J. L. M. Hermens, P. de Voogt, M. H. S. Kraak

**Affiliations:** 1 Institute for Biodiversity and Ecosystem Dynamics (IBED), University of Amsterdam, P.O. Box 94248, 1090 GE Amsterdam, The Netherlands; 2 Institute for Risk Assessment Sciences, Toxicology Division, Utrecht University, P.O. Box 80177, 3508 TD Utrecht, The Netherlands; 3 Ecofide, Singel 105, 1381 AT Weesp, The Netherlands; 4 KWR Watercycle Research Institute, P.O. Box 1072, 3430 BB Nieuwegein, The Netherlands

**Keywords:** Aquatic ecotoxicity, *Daphnia*, Solubility, Anionic surfactants, Alkyl sulfonates, Alkyl sulfates, Alkyl carboxylates

## Abstract

In order to develop models that can predict the environmental behavior and effects of chemicals, reliable experimental data are needed. However, for anionic surfactants the number of ecotoxicity studies is still limited. The present study therefore aimed to determine the aquatic ecotoxicity of three classes of anionic surfactants. To this purpose we subjected daphnids (*Daphnia magna*) for 48 h to alkyl carboxylates (C_x_CO_2_^−^), alkyl sulfonates (C_x_SO_3_^−^), and alkyl sulfates (C_x_SO_4_^−^) with different carbon chain lengths (x). However, all surfactants with x > 11 showed less than 50% immobility at water solubility. Hence, EC_50_ values for only few surfactants could be gathered: C_9_CO_2_^−^ (16 mg L^−1^), C_11_CO_2_^−^ (0.8 mg L^−1^) and C_11_SO_4_^−^ (13.5 mg L^−1^). Data from these compounds showed an increase in ecotoxicity with a factor 4.5 per addition of a hydrocarbon unit to the alkyl chain, and a factor 20 when replacing the sulfate head group by a carboxylate head group. Unfortunately, we could not test carboxylates with a broader variety of chain lengths because solubility limited the range of chain length that can be tested.

Numerous new organic chemicals are produced yearly for application in industry and consumer products (CEFIC 2014). For environmental risk assessment of new and existing chemicals, an understanding of their environmental behavior and effects is required, but for anionic surfactants the number of ecotoxicity studies is still limited. For the development of predictive models such as quantitative structure–activity relationships (QSARs) for surfactants, more experimental data for these group of compounds are therefore needed. Although some toxicity tests on surfactants have been performed thus far (Schüürmann 1990; Roberts 1991; Versteeg et al. 1997; Wong et al. 1997; Dyer et al. 2000; Roberts and Costello 2003; Boeije et al. 2006; Hodges et al. 2006; Qi et al. 2011), the data is still too limited to compare the effect between different surfactant groups (i.e., surfactants with different head group structures), certainly for anionic surfactants. In this study, we therefore focused on generating aquatic ecotoxicity data for anionic surfactants from three different surfactant groups.

Anionic surfactants are high production volume chemicals which are present in many consumer products and consequently also in the environment (Sanderson et al. 2006; CEFIC 2014). Their amphiphilic and electrostatic properties make them very efficient compounds for the detergent industry. At the same time, these properties result in a very different environmental behavior compared to e.g. neutral organic compounds (Jones et al. 2003; Guo and Gaiki 2005). Unlike for common neutral organic pollutants, their accumulation and potential effects can therefore not always be correlated with predicted octanol–water partition constants (log *K*_ow_) (Tolls and Sijm 1995).

The ecotoxicity of organic compounds (quantified by the concentration causing a 50% effect; EC_50_ value) is usually determined in standardized *Daphnia magna* acute ecotoxicity tests according to OECD guideline 202 (OECD 2004). For some surfactants within a specific surfactant group (i.e., homologues sharing the same head group), toxicity is observed to increase with increasing alkyl chain length due to increased hydrophobicity (Roberts 2000; Roberts et al. 2013; Barmentlo et al. 2015). At the same time, hydrophobicity affects the bioavailability of surfactants by decreasing the solubility, but also by increasing sorption to other phases (Pittinger et al. 1989). Bioavailability of anionic surfactants is also influenced by the electrostatic characteristics of the head group, which can result in ion-pairing with divalent inorganic cations (e.g., Ca^2+^ or Mg^2+^) (Rodriguez et al. 2001; Yan et al. 2010). The standard medium in the *D. magna* toxicity test (OECD 2004) contains a relatively high total ionic strength that includes divalent cations and solubility problems can therefore be expected for some surfactants. The determination of EC_50_ values for (ionic) compounds with a low solubility using OECD guideline 202 can therefore be challenging. However, since experimental data for anionic surfactants is still much needed, the aim of the present study was to employ the standardized *D. magna* ecotoxicity test to determine the aquatic ecotoxicity of three classes of anionic surfactants: alkyl carboxylates, alkyl sulfonates, and alkyl sulfates.

## Materials and Methods

All test compounds had a typical surfactant structure containing a hydrophobic alkyl chain and a hydrophilic ionized head group. Sodium salts of linear alkyl sulfates (C_x_SO_4_^−^; with alkyl chain lengths C_11_, C_13_, C_15_ and C_16_) and linear alkyl sulfonates (C_x_SO_3_^−^; C_11_, C_13_, C_14_ and C_15_) were obtained from Research Plus (South Plainfield, NJ). Sodium salts of linear alkyl carboxylates (C_x_CO_2_^−^; C_9_, C_11_, C_13_, C_14_, and C_15_) were obtained from Sigma-Aldrich, (Zwijndrecht, The Netherlands). All organic compounds had purities higher than 98%. Ammonium acetate was purchased from Sigma-Aldrich. Methanol was obtained from Biosolve (Valkenswaard, The Netherlands). Ultrapure water was obtained from a Millipore water purification system (resistivity > 18 MΩ/cm, Merck Chemicals, Amsterdam, The Netherlands).

The daphnid *D. magna* Straus was selected as test organism to determine the aquatic ecotoxicity of surfactants. Juvenile daphnids (clone 4) aged < 24 h were obtained from adults between 2 and 3 weeks old. Continuous cultures were maintained in Elendt M4 medium and fed with the alga *Chlorella vulgaris*. At regular intervals (about every 3 months), acute toxicity tests were performed with the reference toxicant K_2_Cr_2_O_7_ to check whether the sensitivity of the daphnids culture was within the limits (EC_50_, 24 h = 0.6–2.1 mg L^−1^) as set by the guideline (OECD 2004). The medium used in the toxicity experiments consisted of the standard OECD medium that was prepared according to OECD guideline 202, containing 266 mg L^−1^ CaCL_2_·2H_2_O, and 112 mg L^−1^ MgSO_4_·7H_2_O. Concentrations of KCl and NaHCO_3_ were 5 and 65 mg L^−1^ respectively (OECD 2004). The test media was buffered to pH 7 ± 0.3 with NaOH (66 mg L^−1^) and 3-(*N*-morpholino)propanesulfonic acid (MOPS; 1.046 g L^−1^).

The *D. magna* were exposed to the selected compounds in 48 h immobility tests (OECD 2004). Per experiment five test concentrations, a solvent control (0.25% methanol without the test compound) and a control were tested with four replicates per treatment. Each replicate consisted of a glass tube filled with 20 mL of test solution, spiked with 50 µL (0.25% of total volume) methanol containing the test compound. The tubes were randomly distributed in a climate controlled fume hood (20 ± 1°C), with a light–dark regime of 16:8 h. The experiment was started by introducing five neonates (younger than 24 h) into each tube. After 48 h, the number of animals not responding to stimulation was scored. Hardness, oxygen concentration, temperature and pH were measured at the start and the end of the experiments and were within the range prescribed by OECD guideline 202 (OECD 2004). The concentration of the test compounds was analyzed by extracting a 200 µL water sample from each replicate at the start and the end of the experiment, an injection standard was added and the sample was subsequently diluted with 750 µL of methanol and stored in a freezer (− 18°C) until chemical analysis.

All anionic surfactants were detected with a triple quadrupole mass spectrometer (MDS SCIEX API 3000 MS/MS System from Applied Biosystems, Bleiswijk, The Netherlands) with a Turbo Ion spray source operated at 400°C. A solvent delay switch (Da Vinci, Rotterdam, The Netherlands) was used to prevent introduction of inorganic constituents from water samples into the MS. Chromatograms were integrated with Analyst 1.4.2 (Applied Biosystems). Concentration–response relationships and the corresponding 48 h EC_50_ values were calculated according to Haanstra et al. (1985) by fitting a logistic curve (Eq. 1) to the percentage of mobility (100% − immobilization) versus the surfactant concentration in the water phase.

1 $$y(x)=\frac{C}{{1+{e^b}({{\log }_{10}}x - {{\log }_{10}}a)}}$$

where *y*(*x*) is the mobility at concentration *x* (in %), *a* is the EC_50_ value (in mg L^−1^), *b* is the slope of the curve, *c* is *y*(0) which equals the average mobility of the control and *x* is the surfactant concentration in water (in mg L^−1^). Data analyses were performed with SPSS software (IBM Corp 2013) and Graphpad Prism Version 7.0 (GraphPad Software 2017).

## Results and Discussion

A total of 14 surfactants with varying alkyl chain lengths from three surfactant groups (alkyl sulfates, alkyl sulfonates, and alkyl carboxylates) were tested. Due to their hydrophobicity and electrostatic charge, anionic surfactants with long alkyl chains often poorly dissolve in water containing inorganic cations. We therefore decided to first test the effect of saturated water solutions at maximum aqueous solubility (*S*_w_) on the daphnids. To this end we stirred an excess of compound for 48 h in standard OECD medium under the standard conditions of the *D. magna* toxicity tests (OECD 2004). For the compounds that caused more than 50% immobility of the daphnids at *S*_w_, a concentration range was tested in order to obtain concentration–response relationships and to derive EC_50_ values.

We were unable to dissolve alkyl sulfonates (C_x_SO_3_^−^) in the OECD medium at sufficiently high concentrations to cause any effect. This may have been a result of the presence of (divalent) cations in the aqueous phase. Cations are known to affect the hydration of anionic surfactants and often lowers their critical micelles concentration (CMC) (Yan et al. 2010). Divalent cations such as Ca^2+^ and Mg^2+^ can furthermore form ion pairs containing two surfactant monomers and one divalent cation, or form bridges between monomers and charged sites on sorbents (Haftka et al. 2015). For the alkyl carboxylates (C_x_CO_2_^−^) and the alkyl sulfates (C_x_SO_4_^−^), compounds with an alkyl chain longer than C_11_ were badly soluble in the OECD medium and showed less than 50% immobility at *S*_w_. Hence, no further ecotoxicity tests were performed for these compounds.

**Fig. 1:**
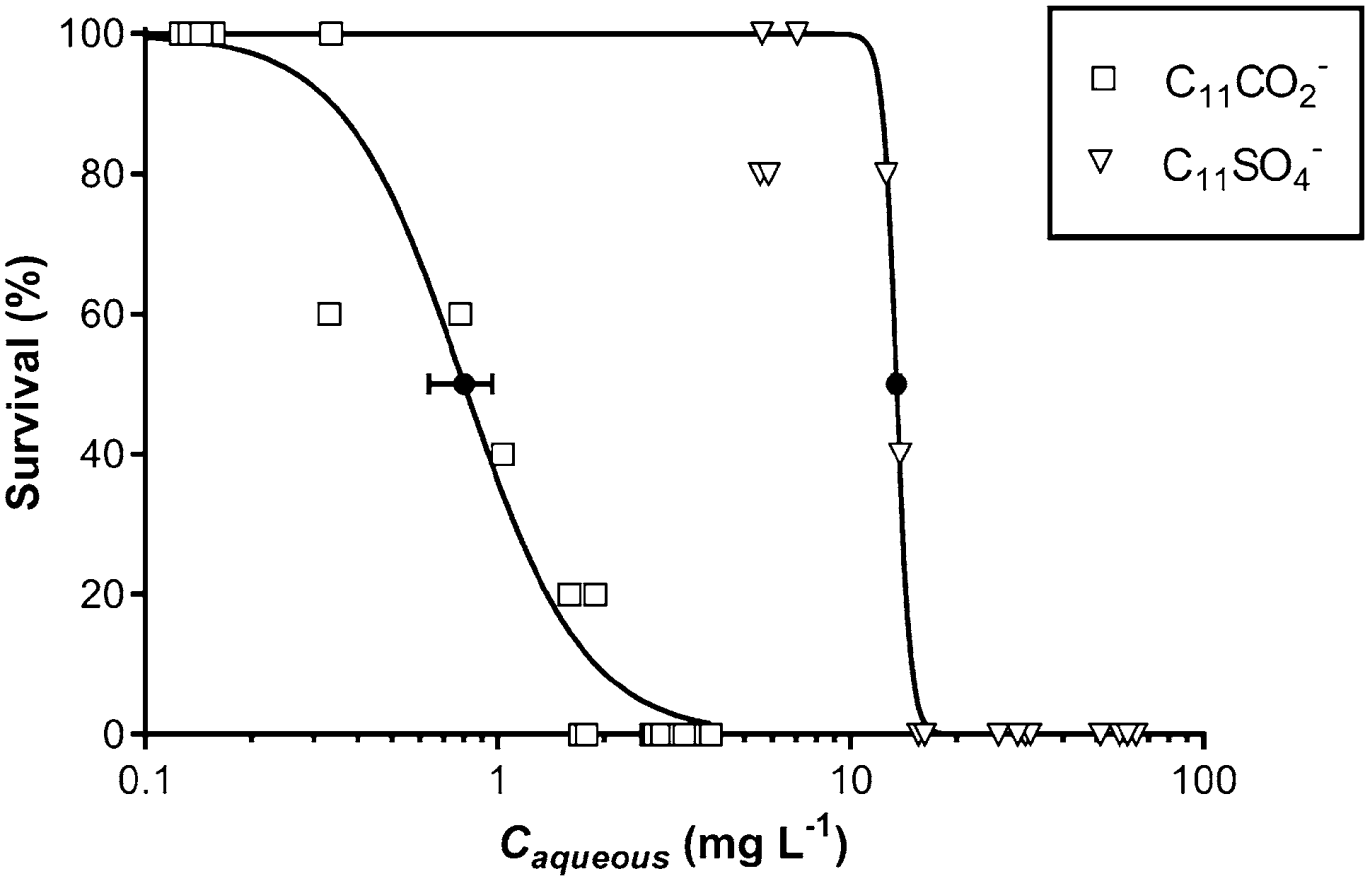
Effect of head group on ecotoxicity of C_11_SO_4_^−^ and C_11_CO_2_^−^ to *Daphnia magna* after 48 h of exposure. Both dose–response curves were calculated according to Haanstra et al. (1985). The EC_50_ concentrations are plotted with their 95% confidence intervals as solid black symbols (the 95% confidence interval of C_11_SO_4_^−^ is too small to be seen)

Because of the solubility problems of the tested compounds in the OECD medium, EC_50_ values for only few anionic surfactants could be generated: C_9_CO_2_^−^, C_11_CO_2_^−^ and C_11_SO_4_^−^. Because one pair of these surfactants contains equal alkyl chain lengths and different surfactant head groups (C_11_CO_2_^−^ and C_11_SO_4_^−^), and another pair (C_9_CO_2_^−^ and C_11_CO_2_^−^) differs in chain length with equal head group, we had two single opportunities to evaluate the effect of head group structure and alkyl chain length on the toxicity of the anionic surfactants. However, note that these interpretations are based on only a single pair of surfactants. For C_9_CO_2_^−^, C_11_SO_4_^−^ and C_11_CO_2_^−^ analyzed concentrations were respectively ± 10%, ± 10% and ± 30% lower compared to nominal concentrations. During the 48 h *D. magna* toxicity experiments 100% control survival was recorded. From the dose–response curve of C_11_SO_4_^−^ an EC_50_ value of 13.5 mg L^−1^ was derived (95% CI 13.2–13.8 mg L^−1^) (Fig. 1). We were unable to find any EC_50_ values of C_11_SO_4_^−^ in literature as most studies focused on C_12_SO_4_^−^. Persoone et al. (1989) reported an EC_50_ value of 9.6 mg L^−1^ for C_12_SO_4_^−^ in a *D. magna* 24 h toxicity test and Dyer et al. (1996) found an EC_50_ value of 5.5 mg L^−1^ in a 48 h *Ceriodaphnia dubia* toxicity test (comparable sensitivity to *D. magna* (Versteeg et al. 1997)). Both values are in line with our data for C_11_SO_4_^−^, as toxicity generally increases from 24 to 48 h exposure and an EC_50_ value of 5.5 mg L^−1^ is close to the expected EC_50_ concentration increase when a hydrocarbon (–CH_2_–) unit is added to the alkyl chain of C_11_SO_4_^−^ (see next paragraph). The dose–response curve of C_11_CO_2_^−^ provided an EC_50_ concentration of 0.80 mg L^−1^ (95% CI 0.7–0.9 mg L^−1^) (Fig. 1). Toxicity data for *D. magna* are scarce for C_11_CO_2_^−^, a 36x higher EC50 value (EC_50_ = 29 mg L^−1^) was reported by Lundahl and Cabridenc (1978) in a 24 h ecotoxicity test, and an EC_50_ value of 1.3 mg L^−1^ was reported by the European Chemical Agency (2014). While, we were unable to acquire the exact experimental details of the toxicity test of Lundahl and Cabridenc (1978), their analysis was performed using the Methylene Blue Active Substance (MBAS) essay which is meanwhile retracted as a standard method by ASTM.

Comparing the dose–response curves and EC_50_ values for C_11_SO_4_^−^ and C_11_CO_2_^−^ shows that the head group has a significant effect on ecotoxicity (Fig. 1). The alkyl chains of both compounds are of the same length and the effect of hydrophobicity is subsequently similar (Hammer et al. 2017). Therefore, the difference in EC_50_ values is likely a result from the different molecular properties of the surfactant head groups (SO_4_^−^ vs. CO_2_^−^). Besides the head group structure, the most notable distinction between the properties of these two surfactant groups is the difference in p*K*_a_ [4.8 for C_x_CO_2_^−^ (Haynes 2015), and − 3.6 for C_x_SO_4_^−^ (COSMOlogic 2015)]. The p*K*_a_ value is partly a result of the charge distribution over a molecule and shows what fraction of the compound is in the ionic form at certain pH. While these compounds are in the OECD medium both for > 99% present in their ionic (de-protonated) form, the difference charge distribution between both molecules still affects their behavior in the aqueous phase and their interaction with other phases. For example, alkyl carboxylates are much better hydrated than alkyl sulfates (Vlachy et al. 2009), which also affects their electrostatic interaction with sorbents (Rabin and Stillian 1994). Furthermore, the difference in charge distribution may affect the uptake of the anionic surfactants in cell membranes due to their zwitterionic properties (Scherer and Seelig 1989). Badly hydrated compounds are usually more affected by local charges and have more difficulty to partition into membranes than well hydrated compounds (Jing et al. 2009; Roberts et al. 2013). The C_11_CO_2_^−^ surfactant may therefore partition more effectively into cell membranes of the daphnids compared to C_11_SO_4_^−^ which explains why alkyl carboxylates were approximately 20 times more toxic compared to their sulfated counterparts.

**Fig. 2:**
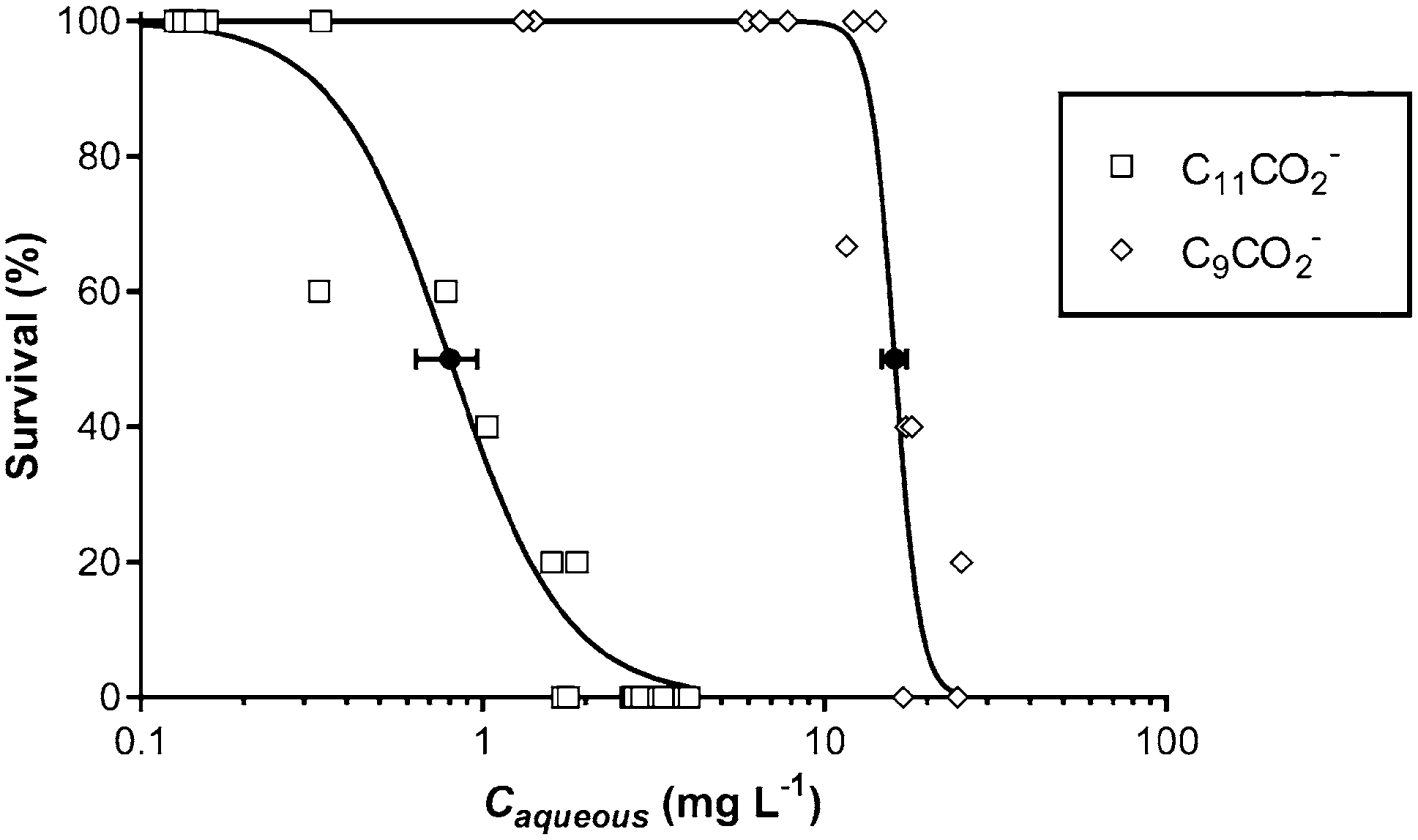
Effect of alkyl chain length on ecotoxicity of C_11_CO_2_^−^ and C_9_CO_2_^−^ on *Daphnia magna* after 48 h of exposure. Both dose–response curves were calculated according to Haanstra et al. (1985). The EC_50_ concentrations are plotted with their 95% confidence intervals as solid black symbols

The effect of the alkyl chain length on surfactant toxicity was studied by comparing the EC_50_ values of C_9_CO_2_^−^ and C_11_CO_2_^−^. The dose–response curve for C_9_CO_2_ showed an EC_50_ concentration of 16.0 mg L^−1^ (95% CI 14.8–17.3 mg L^−1^), see Fig. 2. Just like for the previously discussed surfactants, literature data on the toxicity of C_9_CO_2_^−^ to *D. magna* is inconsistent and details about the experimental setup were difficult to obtain. We were able to find two EC_50_ concentrations from literature: first, again a very high EC_50_ concentration of 135 mg L^−1^ from a 24 h *D. magna* toxicity test by Lundahl and Cabridenc (1978). Second, a reported EC_50_ value of 16 mg L^−1^, which is equal to our experimentally derived EC_50_ value and originates from a report of the European Chemical Agency (2013). The results from Lundahl and Cabridenc are questionable (see previous paragraph) and both studies lack experimental details about medium composition and only mention the duration of the tests. Toxicity between C_11_ and C_9_ carboxylate differed with a factor of ∼ 23 compared (Fig. 2), which is a factor of ∼ 4.5 per hydrocarbon unit added to the alkyl chain. This is somewhat higher than the increments found for other surfactant groups in previous studies [between 2.4 and 3.4 (Lundahl and Cabridenc 1978; Maki and Bishop 1979; Hodges et al. 2006)]. An increase in the alkyl chain length increases the hydrophobicity of the compound and thus increases the sorption to the membrane lipid (Könnecker et al. 2011). At longer alkyl chain lengths (> C_11_) the toxicity is expected to further increase, but this effect is not detectible using the *D. magna* toxicity test due the low solubility of the compounds in the OECD medium. The factor ~ 4.5 increase in toxicity with addition of a carbon atom to the alkyl chain is based on only two chemicals. This data set is limited and could be regarded as a shortcoming of the study. Unfortunately, we could not test more compounds because of the solubility problems (limits) of the longer chain carboxylates in the calcium rich test medium of the Daphnia test. Another test organism that requires another medium composition (less calcium) could avoid this shortcoming.

The main reason why ecotoxicity could not be detected for most of the test compounds probably lies in the presence of cations in the aqueous solution of the *D. magna* tests, which can affect the solubility and bioavailability of anionic surfactants. In an attempt to generate more ecotoxicity data, we decided to change the composition of the original OECD medium and study the effect of divalent cation concentration on the ecotoxicity of C_9_CO_2_^−^ and C_11_CO_2_^−^. To this end, four different media were prepared with different concentrations of Ca^2+^ and Mg^2+^, while maintaining original Ca^2+^:Mg^2+^ ratio (Naddy et al. 2002). A concentration of Ca^2+^ of 10 mg L^−1^ was selected as the lowest concentration, because lower concentrations will affect with *D. magna* survival (Hessen et al. 2000). The highest concentration of Ca^2+^ tested was 80 mg L^−1^, conform with the original OECD guideline 202. The resulting EC_50_ concentrations varied slightly, but did not differ significantly between medium compositions. Hence, the medium with the lowest ionic strength may already contain enough cations to cause precipitation of anionic surfactants.

The *D. magna* toxicity test is a well-accepted and standardized toxicity test which has generated ecologically relevant toxicity data for many organic compounds. However, the medium proposed in the OECD guideline for *D. magna* is of high ionic strength and this can result in solubility problems for compounds that are already barely soluble in water and for compounds that maintain an electrostatic charge (Waaijers et al. 2013). The *D. magna* toxicity test appeared unable to produce reliable results for most of the surfactants tested in this study. For hazard assessment purposes of anionic surfactants, alternative approaches should therefore be investigated that either exclude the influence of divalent cations present in the test medium or endpoints should be selected that are affected at concentrations below the aqueous solubility of the surfactants. Furthermore, because anionic surfactants are known to have an affinity for soil surfaces and organic matter (Rico–Rico 2009) toxicity tests that include sediment living organisms (e.g. *Lumbriculus variegatus* or *Chironomus riparius*) may be more suitable for the production of toxicological endpoint data. Despite the obstacles that occurred with anionic surfactants during the *D. magna* tests, we were able to determine the effect of surfactant alkyl chain length and head group composition on the aquatic ecotoxicity of a select group of anionic surfactants. However, these interpretations were based on only a single pair of surfactants.
